# Carcinogenic Air Toxics Exposure and Their Cancer-Related Health Impacts in the United States

**DOI:** 10.1371/journal.pone.0140013

**Published:** 2015-10-07

**Authors:** Ying Zhou, Chaoyang Li, Mark A. J. Huijbregts, M. Moiz Mumtaz

**Affiliations:** 1 Environmental Health Tracking Branch, Division of Environmental Hazards and Health Effects, National Center for Environmental Health, Centers for Disease Control and Prevention, Atlanta, Georgia, United States of America; 2 Department of Environmental Science, Institute for Water and Wetland Research, Radboud University Nijmegen, NL-65OO GL, Nijmegen, The Netherlands; 3 Computational Toxicology Laboratory, Division of Toxicology and Human Health Sciences, Agency for Toxic Substances and Disease Registry, Centers for Disease Control and Prevention, Atlanta, Georgia, United States of America; Utah State University, UNITED STATES

## Abstract

Public health protection from air pollution can be achieved more effectively by shifting from a single-pollutant approach to a multi-pollutant approach. To develop such multi-pollutant approaches, identifying which air pollutants are present most frequently is essential. This study aims to determine the frequently found carcinogenic air toxics or hazardous air pollutants (HAPs) combinations across the United States as well as to analyze the health impacts of developing cancer due to exposure to these HAPs. To identify the most commonly found carcinogenic air toxics combinations, we first identified HAPs with cancer risk greater than one in a million in more than 5% of the census tracts across the United States, based on the National-Scale Air Toxics Assessment (NATA) by the U.S. EPA for year 2005. We then calculated the frequencies of their two-component (binary), and three-component (ternary) combinations. To quantify the cancer-related health impacts, we focused on the 10 most frequently found HAPs with national average cancer risk greater than one in a million. Their cancer-related health impacts were calculated by converting lifetime cancer risk reported in NATA 2005 to years of healthy life lost or Disability-Adjusted Life Years (DALYs). We found that the most frequently found air toxics with cancer risk greater than one in a million are formaldehyde, carbon tetrachloride, acetaldehyde, and benzene. The most frequently occurring binary pairs and ternary mixtures are the various combinations of these four air toxics. Analysis of urban and rural HAPs did not reveal significant differences in the top combinations of these chemicals. The cumulative annual cancer-related health impacts of inhaling the top 10 carcinogenic air toxics included was about 1,600 DALYs in the United States or 0.6 DALYs per 100,000 people. Formaldehyde and benzene together contribute nearly 60 percent of the total cancer-related health impacts. Our study shows that although there are many carcinogenic air toxics, only a few of them affect public health significantly at the national level in the United States, based on the frequency of occurrence of air toxics mixtures and cancer-related public health impacts. Future research is needed on their joint toxicity and cumulative health impacts.

## Introduction

Our ambient environment contains naturally occurring chemicals and xenobiotics introduced by human activities. For example, hazardous air pollutants can originate from anthropogenic sources, including mobile sources (e.g., cars, trucks, buses), stationary sources (e.g., factories, refineries), indoor sources (e.g., building materials, cleaning solvents), as well as natural sources (e.g., volcanic eruptions, forest fires). Human exposures to environmental chemicals and often their mixtures are extremely complex, involving a multitude of chemicals and through various exposure scenarios. Defining a subset of chemicals representative of a larger group, such as the priority list of the top 275 hazardous substances found at waste sites [1], has been used to reduce complex problems to more manageable ones. This concept has been advanced further to identify priority mixtures too [2]. When assessing human-health risk, this concept can help prioritize chemical mixtures for toxicological research based on potential exposure and toxicity to human populations. Several attempts have been made to identify environmental mixtures near hazardous waste sites to help advance the joint-toxicity methods-development process [2,3]. For example, Fay and Mumtaz (1996) conducted a frequency-of-occurrence analysis of chemicals in the air at 1,188 U.S. hazardous waste sites and reported that the most frequently found binary combination was benzene and toluene; the most common ternary combination was benzene, perchloroethylene, and trichloroethylene.

The U.S. Environmental Protection Agency (EPA) defines two major types of air pollutants for regulatory purposes in the United States—criteria air pollutants and hazardous air pollutants (HAPs). EPA has set National Ambient Air Quality Standards (NAAQS) for six common criteria pollutants—particulate matter (PM), ozone, sulfur dioxide, nitrogen dioxide, carbon monoxide, and lead [4]. HAPs, also known as air toxics are known or suspected to cause cancer or other serious health effects, such as neurological, reproductive, respiratory problems [5]. In 1990, the U.S. Congress identified 187 chemicals and compound groups as HAPs under Section 112 of the Clean Air Act [5]. Historically HAPs have been a focus for source-specific emissions standards.

EPA has completed four National-Scale Air Toxics Assessments (NATA) (1996, 1999, 2002, and 2005) that characterize the nationwide chronic cancer-risk estimates and non-cancer hazards from inhaling air toxics. The 2005 NATA assessment includes four sequential steps (1) compiling a national inventory of air toxics emissions from outdoor sources; (2) Estimating ambient outdoor concentrations of the emitted air toxics across the United States at each census tract by using air quality models, which are mathematical equations that use emissions, meteorological, and other information to simulate the behavior and movement of air toxics in the atmosphere; (3) estimating long-term population exposures to these air toxics via inhalation by the exposure ratio approach, which relies on ambient to exposure concentration ratios developed in previous NATA assessments for each combination of source type, census tract, and air toxic; and (4) characterizing potential public health risks due to inhaling air toxics including both cancer and non-cancer effects [5]. The lifetime cancer risk associated with exposure to a single air pollutant is estimated by multiplying an average estimated long-term exposure concentration at each census tract by the corresponding inhalation unit risk estimate (URE) for that pollutant as follows [5]:

$${Risk}_{ij}={EC}_{ij}\times {URE}_{i}$$ (1)

Risk_ij_ = estimated incremental lifetime cancer risk for an individual in census tract j as a result of exposure to air toxic i, which is unitless number expressed as a probability;

EC_ij_ = estimate of 70-year average inhalation exposure concentration in census tract j for air toxic i, in units of μg/m^3^;

URE_i_ = the corresponding inhalation unit risk estimate for air toxic i, in units of (μg/m3)^-1^.

Note that EC_ij_ varies by air toxic and census tract and it is the output from the step 2 and 3 of the NATA assessment discussed above. In addition, it is worth noting that the toxicity values, i.e., URE, is the upper-bound excess lifetime cancer risk estimated to result from continuous exposure to an agent at a concentration of 1 μg/m^3^ in air. As a result, the “true” cancer risks are likely below the NATA estimates, though the likelihood varies among the different pollutants [5].

Among the 187 Clean Air Act air toxics, the 2005 NATA assessment provides cancer risk results for 81 air toxics that have emissions data and chronic health toxicity values available. The 2005 NATA reported cancer risk as a statistical probability of an individual to develop cancer over one’s lifetime at both county and census-tract levels for these 81 carcinogenic air toxics (Table A in S1 File). It estimated that collectively the inhalation of these 81 air toxics simultaneously at the predicted concentrations over a lifetime of 70 years would cause ≈ 1 in every 20,000 persons nationwide to contract cancer in their lifetime, corresponding to a national average cancer risk of ≈ 50 in a million.

There are some limitations to NATA estimates, such as it focuses on inhalation and does not reflect all pathways of exposure; it reflects only compounds released into the outdoor air as no indoor sources were included; and it might not accurately capture sources that have episodic emissions [5]. NATA results have been applied by other researchers in different settings, such as comparing and ranking the relative health risks of air toxics in Houston, Texas [6], and examining the relationship between HAP exposure and neighborhood socioeconomic deprivation [7].

Our study aims to focus on carcinogens with cancer risks reported in NATA 2005 and to determine the frequency of occurrence of various carcinogen mixtures in the air across the United States as well as to analyze the difference between urban and rural areas. In addition, we also aim to estimate the cancer-related public health impacts of the top carcinogenic air toxics by using disability-adjusted life years (DALYs), which incorporates the magnitude of the cancer risk for different air toxics and severity of the corresponding type of cancer risks. These results would allow public health professionals to develop multi-pollutant control strategies, which could be more efficient in protecting public health than a single pollutant approach [8,9].

## Methods

### Frequencies of air toxics mixtures based on cancer risk

To identify the chemical mixtures that most frequently cause cancer, we first determined the number of census tracts with NATA 2005 cancer risk greater than one in a million for each pollutant. For the pollutants with cancer risk greater than one in a million in more than 5% of the census tracts and with national average cancer risk > one in a million, we estimated the frequencies of their binary and ternary combinations. The number of combinations was calculated by $\frac{n!}{r!(n-r)!}$, where n is the number of pollutants; r is the levels of combination desired, which was equal to 2 for binary pairs and 3 for ternary combinations in our analysis.

#### Sensitivity analysis

To test the robustness of our findings as well as to allow for further prioritization so as to select chemical mixtures with the highest cancer risk, we repeated the previous calculation by increasing the cancer risk threshold to three in a million and conducted a sensitivity test.

In addition, as part of the sensitivity analysis, we analyzed urban and rural areas separately in terms of the frequencies of air toxics with cancer risk greater than one in a million, their binary pairs, and ternary combinations. We used the same urban definition in the NATA county-level dataset. A county is considered urban if it either includes a metropolitan statistical area with a population greater than 250,000 or the U.S. Census Bureau designates more than 50% of the population as urban in the 2000 U.S. census data [5]. The remaining counties are considered rural. We assigned all the census tracts in a given county the same urban or rural designation as the county. Based on this definition, the dataset contains 1,162 urban counties (which include 53,438 census tracts and ≈ 233 million people) and 2,061 rural counties (which include 12,597 census tracts and 52 million people).

**Fig 1:**
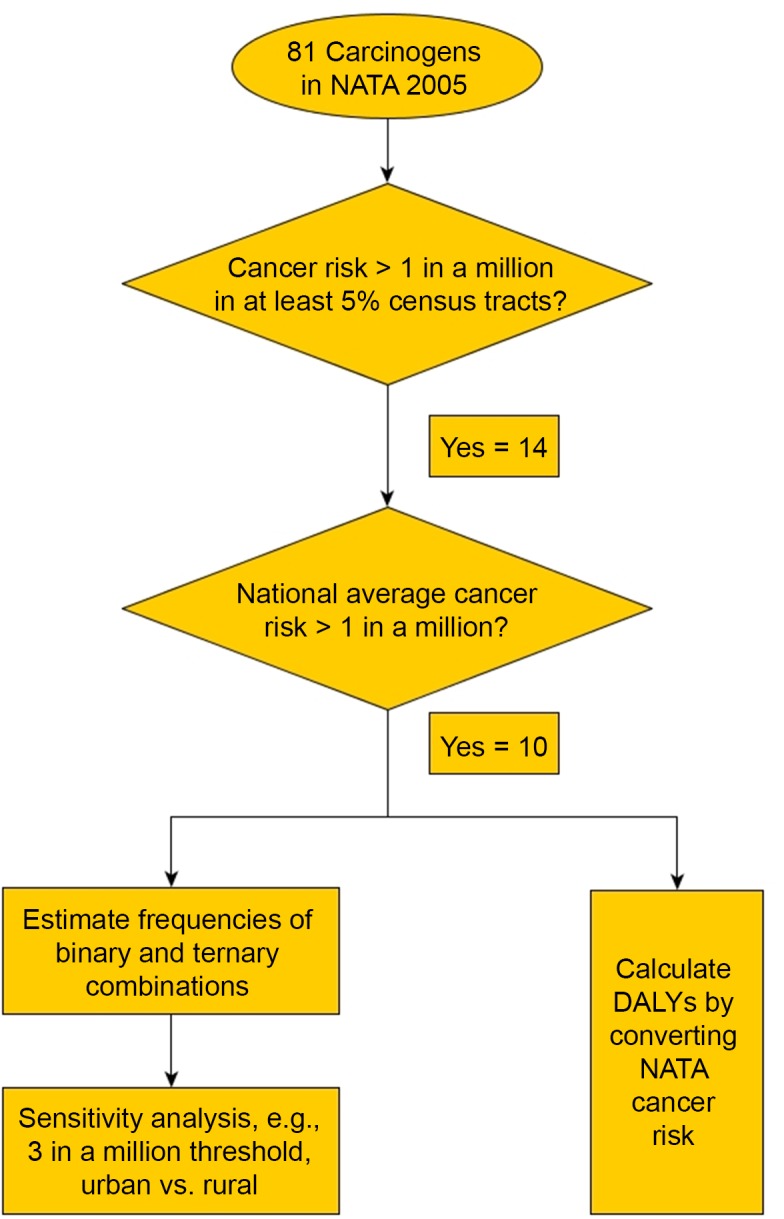
Data analysis flowchart.

Fig 1 provides a summary of the different steps in the data analysis. The left-hand side of the Figure shows the components related to the frequencies of air toxics mixtures discussed above.

### Cancer-related Health impacts of air toxics mixtures

Disability-adjusted life years (DALYs) quantify overall disease burden due to both mortality and morbidity by summing years of life lost due to premature mortality and years lived with disability [10]. One DALY can be thought of as one lost year of "healthy" life due to ill-health, disability or early death related to the health outcome of concern, i.e., cancer in this analysis.

To quantify cancer-related annual health impacts from exposure to each air toxic in this study, we converted lifetime cancer risk reported in NATA 2005 to DALYs using the following equation:

$${DALYs}_{ij}={\text{cancer risk}\;}_{ij}\times {pop}_{j}\times \frac{DALYe}{70}$$ (2)

DALYs_ij_ is the Disability-adjusted life years for air toxic i in census tract j;

cancer risk_ij_ is the estimated incremental lifetime cancer risk for an individual as a result of exposure to air toxic i (unitless number expressed as a probability) in census tract j reported in NATA 2005;

pop_j_ is the population in census tract j based on 2000 U.S. census data [5];

*DALY*_*e*_ is the severity factor or years of “healthy” life lost per cancer case corresponding to cancer type e;

“70” is the commonly used life expectancy of 70 years, which is a default value in environmental risk assessment. Cancer risks are presented as lifetime risks in NATA 2005, meaning the risk of developing cancer as a result of inhalation exposure to each air toxic over a lifetime of 70 years. Thus, 70 is used in the denominator to derive the annual cancer-related health impacts due to exposure to air toxics.

Note that to determine the cancer type for each of the 10 most frequently found air toxics with national average cancer risk greater than one in a million, we reviewed toxicity databases such as Integrated Risk Information System (IRIS) [11], 13th Edition of Report on Carcinogens by National Toxicology Program [12], the Toxicity Criteria Database of California’s Office of Environmental Health Hazard Assessment (OEHHA) [13]. Table 1 shows the cancer type for each air toxic. DALY per cancer case for different cancer types were estimated based on Murray and Lopez [14]. We used a similar method for calculating health burden based on Unit Risk factors for carcinogenic substances [15].

**Table 1:** Disability adjusted life years (DALY) per cancer case corresponding to the cancer type for the 10 air toxics with the highest national average cancer risks.

Name of substance	Cancer type	DALY per cancer case (year)
formaldehyde	nose and throat cancer	5.5
carbon tetrachloride	adrenal tumors	8.5
PAHPOM	lung cancer	13.6
chromium VI	lung cancer	13.6
acetaldehyde	nose cancer	5.5
benzene	leukemia	13.7
tetrachloroethylene	liver cancer	14.9
naphthalene	nose cancer	5.5
1,3-butadiene	leukemia	13.7
arsenic	lung cancer	13.6

Note: For carbon tetrachloride, we applied the average DALYs for different types of cancers of 8.5 years, since available information does not provide DALY per case estimate for this type of cancer.

For each air toxic of interest, we repeated calculations of Eq 2 for each census tract. Then we calculated the summary statistics—mean, median, standard deviation, and maximum DALYs for each air toxic.

In addition, we also converted the cancer-related health impacts to DALYs per 100,000 people, by dividing the total cancer-related health impact of each air toxic in the geographic area of interest (e.g., census tract, the entire United States) by the corresponding total population in that area, and then multiply that by 100,000. This measure allows us to remove the influence of population in the area of interest and make comparisons of health impacts across different geographic areas.

## Results

Among the 81 pollutants with cancer risks reported in NATA 2005, 14 have cancer risk greater than one in a million in more than 5% of the census tracts. 10 of them also have national average cancer risk greater than one in a million as shown in Table 2, in the three columns under “Single air toxic”. We focused our analysis on the frequencies of air toxics mixtures and cancer-related health impacts on these 10 air toxics.

**Table 2:** Percent of census tracts with cancer risk greater than one in a million for single air toxic, binary pair, and ternary combination.

Single air toxic			Binary Pair			Ternary Combination		
Rank	Air Toxic	Percent of Census tracts*	Rank	Air Toxic	Percent of Census tracts**	Rank	Air Toxic	Percent of Census tracts***
1	formaldehyde	100.0%	1	carbon tetrachloride, formaldehyde	100.0%	1	acetaldehyde, carbon tetrachloride, formaldehyde	99.9%
2	carbon tetrachloride	100.0%	2	acetaldehyde, carbon tetrachloride	99.9%	2	benzene, carbon tetrachloride, formaldehyde	98.8%
3	acetaldehyde	99.9%	3	acetaldehyde, formaldehyde	99.9%	3	acetaldehyde, benzene, carbon tetrachloride	98.7%
4	benzene	98.8%	4	benzene, formaldehyde	98.8%	4	acetaldehyde, benzene, formaldehyde	98.7%
5	1,3-butadiene	71.0%	5	benzene, carbon tetrachloride	98.8%	5	1,3-butadiene, benzene, formaldehyde	71.0%
6	naphthalene	62.7%	6	acetaldehyde, benzene	98.7%	6	1,3-butadiene, benzene, carbon tetrachloride	71.0%
7	arsenic compounds	53.3%	7	1,3-butadiene, benzene	71.0%	7	1,3-butadiene, carbon tetrachloride, formaldehyde	71.0%
8	chromium compounds	50.9%	8	1,3-butadiene, formaldehyde	71.0%	8	1,3-butadiene, acetaldehyde, benzene	71.0%
9	PAHPOM	40.7%	9	1,3-butadiene, carbon tetrachloride	71.0%	9	1,3-butadiene, acetaldehyde, carbon tetrachloride	71.0%
10	tetrachloroethylene	33.5%	10	1,3-butadiene, acetaldehyde	71.0%	10	1,3-butadiene, acetaldehyde, formaldehyde	71.0%

Note

* Percent of census tracts with cancer risk above one in a million for the corresponding air toxic

** Percent of census tracts with cancer risk above one in a million for both air toxics in the pair

*** Percent of census tracts with cancer risk above one in a million for all three air toxics in the combination.

### Air toxics combinations based on cancer risk

Formaldehyde, carbon tetrachloride, acetaldehyde, and benzene all have cancer risks greater than one in a million for more than 98% of the census tracts. The most frequently occurring binary pairs and ternary mixtures are the various combinations of these four air toxics. See Table 2 for more details.

#### Sensitivity analysis

When we repeated the previous calculations by increasing the cancer-risk threshold to three in a million, carbon tetrachloride was no longer in the top 10 list. No other significant changes were revealed by the sensitivity analysis, except that the frequency of occurrence was lower for the other pollutants, as expected. The binary pair of benzene and formaldehyde ranks first with ≈ 84% of the tracts having cancer risks of greater than three in a million for both pollutants. The pairs of acetaldehyde / formaldehyde, as well as acetaldehyde / benzene, both ranked second with cancer risks of greater than three in a million for both pollutants in ≈ 50% of census tracts. The ternary combination of acetaldehyde / benzene / formaldehyde ranks first with ≈ 50% of census tracts having cancer risks of greater than three in a million for all three pollutants (Table 3).

**Table 3:** Percent of census tracts with cancer risk greater than three in a million for single air toxic, binary pair, and ternary combination.

Single air toxic			Binary Pair			Ternary Combination		
Rank	Air Toxic	Percent of Census tracts*	Rank	Air Toxic	Percent of Census tracts**	Rank	Air Toxic	Percent of Census tracts***
1	formaldehyde	100.0%	1	benzene, formaldehyde	84.2%	1	acetaldehyde, benzene, formaldehyde	51.2%
2	benzene	84.2%	2	acetaldehyde, formaldehyde	56.8%	2	benzene, formaldehyde, naphthalene	25.1%
3	acetaldehyde	56.8%	3	acetaldehyde, benzene	51.2%	3	acetaldehyde, formaldehyde, naphthalene	21.0%
4	naphthalene	25.1%	4	formaldehyde, naphthalene	25.1%	4	acetaldehyde, benzene, naphthalene	21.0%
5	1,3-butadiene	19.1%	5	benzene, naphthalene	25.1%	5	1,3-butadiene, benzene, formaldehyde	19.1%
6	PAHPOM	12.5%	6	acetaldehyde, naphthalene	21.0%	6	1,3-butadiene, acetaldehyde, formaldehyde	17.9%
7	chromium compounds	8.8%	7	1,3-butadiene, formaldehyde	19.1%	7	1,3-butadiene, acetaldehyde, benzene	17.9%
8	tetrachloroethylene	8.5%	8	1,3-butadiene, benzene	19.1%	8	1,3-butadiene, formaldehyde, naphthalene	16.1%
9	arsenic compounds	5.3%	9	1,3-butadiene, acetaldehyde	17.9%	9	1,3-butadiene, benzene, naphthalene	16.1%
10	1,4-dichlorobenzene	3.4%	10	1,3-butadiene, naphthalene	16.1%	10	1,3-butadiene, acetaldehyde, naphthalene	15.6%

Note

* Percent of census tracts with cancer risk above three in a million for the corresponding air toxic

** Percent of census tracts with cancer risk above three in a million for both air toxics in the pair

*** Percent of census tracts with cancer risk above three in a million for all three air toxics in the combination.

As part of the sensitivity analysis, when we analyzed urban and rural areas separately, the top four air toxics (carbon tetrachloride, formaldehyde, acetaldehyde, benzene), are the same for urban and rural areas. All four air toxics had cancer risks greater than one in a million in more than 90% of the census tracts. Tables B and C in S1 File show the frequencies of air toxics with cancer risks greater than one in a million and their binary pairs and ternary combinations in urban and rural areas, respectively. Other than these four air toxics, no other air toxics had cancer risk greater than one in a million in more than 25% of the census tracts in rural areas. By comparison, in urban areas, several additional air toxics (i.e., 1,3-butadiene, naphthalene, arsenic compounds, chromium compounds) had cancer risk greater than one in a million in more than 50% of the census tracts.

### Cancer-related health Impacts in DALYs

The cumulative annual cancer-related health impacts of inhaling the top 10 carcinogenic air toxics included was ≈ 1,600 DALYs in the United States, ≈ a national average of 0.6 DALYs per 100,000 people, or ≈ 0.02 DALY per census tract. Formaldehyde and benzene rank top two in cancer-related health impacts. Together they contribute nearly 60 percent of the total cancer-related health impacts (Table 4).

**Table 4:** Carcinogenic health impacts per year in Disability-adjusted Life Years (DALYs) due to inhalation intake of top 10 carcinogenic air toxics.

		Estimates by census tract				
Rank	Air toxic	Mean DALYs	Median DALYs	Standard Deviation	Maximum	DALYs per 100,000 people
1	formaldehyde	7.7E-03	6.3E-03	5.7E-03	9.5E-02	1.8E-01
2	benzene	6.3E-03	5.0E-03	5.4E-03	1.4E-01	1.5E-01
3	1,3-butadiene	1.7E-03	1.2E-03	1.7E-03	3.9E-02	3.8E-02
4	carbon tetrachloride	1.5E-03	1.4E-03	7.4E-04	1.2E-02	3.5E-02
5	PAHPOM	1.3E-03	6.3E-04	2.1E-03	4.7E-02	3.0E-02
6	acetaldehyde	1.1E-03	9.8E-04	7.2E-04	1.2E-02	2.6E-02
7	chromium compounds	1.1E-03	7.6E-04	1.8E-03	1.3E-01	2.6E-02
8	arsenic compounds	1.1E-03	8.0E-04	1.4E-03	6.3E-02	2.6E-02
9	tetrachloroethylene	1.0E-03	5.1E-04	1.5E-03	3.7E-02	2.3E-02
10	naphthalene	7.9E-04	4.5E-04	1.1E-03	2.6E-02	1.8E-02

**Fig 2:**
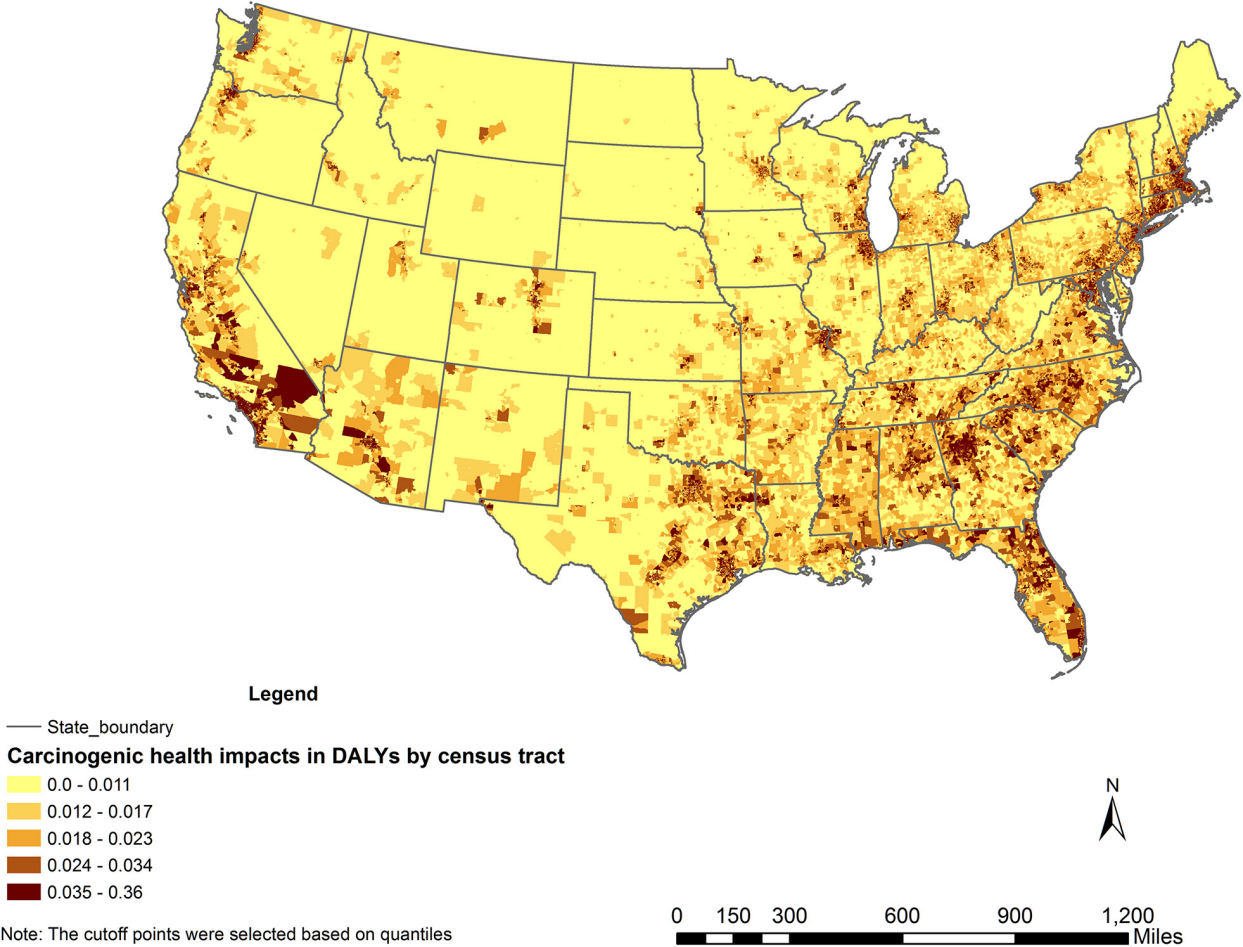
Cumulative carcinogenic health impacts in Disability-Adjusted Life Years (DALYs) per year by census tract caused by inhaling the top 10 carcinogenic air toxics.

In our method for calculating cancer-related health impacts, three factors influence health impacts—(1) estimated incremental lifetime cancer risk reported in NATA 2005, (2) population in each census tract, and (3) the severity factor of the corresponding air toxic’s cancer type. The lifetime cancer risk and population can each vary by several orders of magnitude among different census tracts, while the cancer severity factor we applied vary by a factor of 3. Therefore, we believe the spatial variation in cancer-related health impacts can mainly be attributed to the difference in cancer risk and population density in different census tracts. As shown in Fig 2, the cumulative cancer-related health impacts of the 10 air toxics under study are generally higher in the west coast, the east and the southeast, which is likely a result of the combination of above two factors.

**Fig 3:**
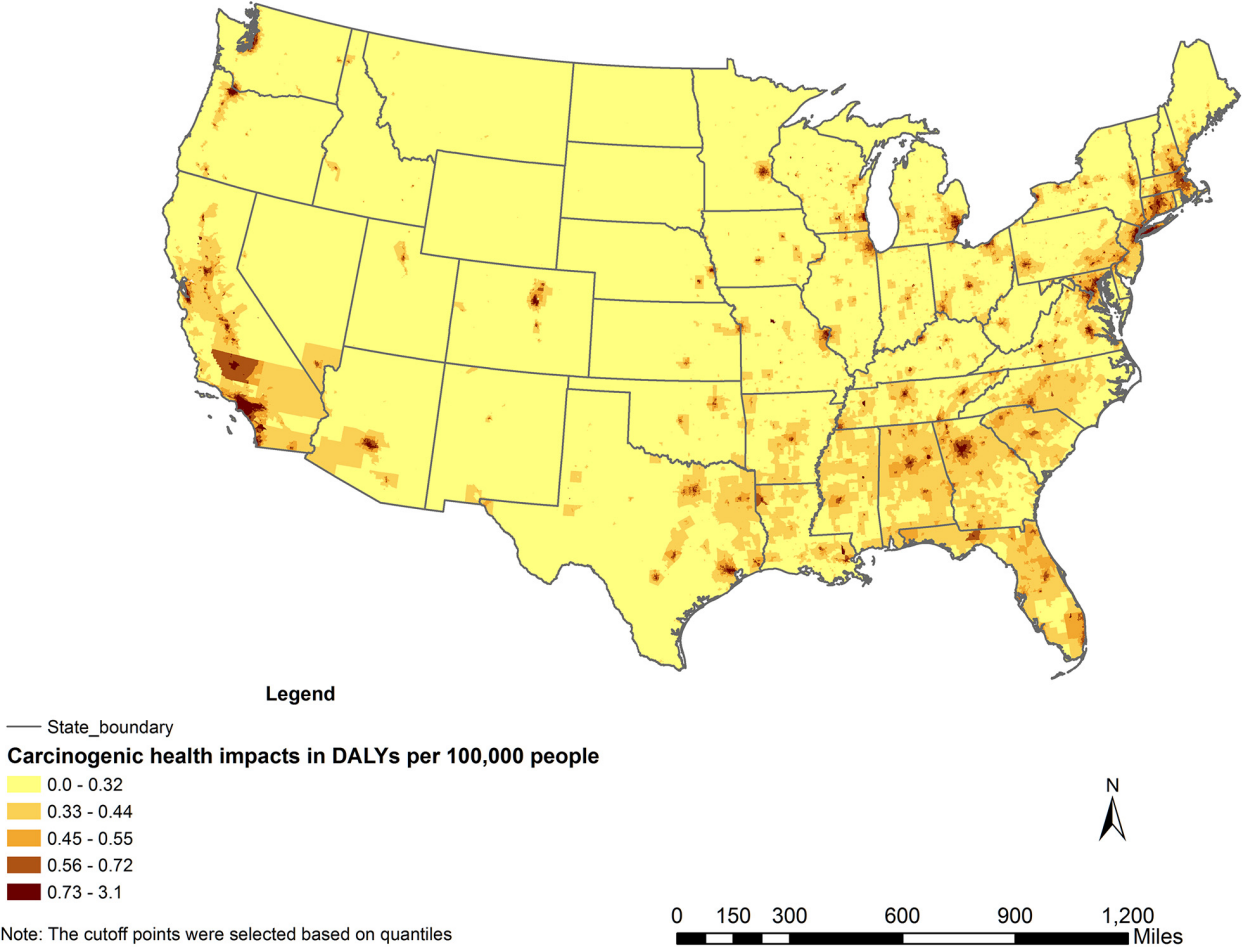
Cumulative carcinogenic health impacts in Disability-Adjusted Life Years (DALYs) per year per 100,000 people caused by inhaling the top 10 carcinogenic air toxics.

In Fig 3, by using DALYs per year per 100,000 people, we removed the influence of the population factor in different census tracts. Comparing with Fig 2, the spatial variation of the cancer-related health impacts is significantly reduced. The remaining variance is mainly due to the variation in incremental lifetime cancer risk reported in NATA 2005.

In addition to the cumulative cancer-related health impacts shown in Figs 2 and 3, Figure A in S1 File also shows the annual carcinogenic health impacts of formaldehyde in DALYs by census tract. Figure B in S1 File shows the annual carcinogenic health impacts of benzene in DALYs by census tract.

## Discussion

Environmental contaminants such as HAPs are present in and around us. Even though we are exposed to multiple chemicals simultaneously, their risks are often assessed as individual chemicals. For public health and environmental protection, the big picture of these exposures has to be clearly understood. Our analysis has revealed that the most frequently occurring binary and ternary mixtures are a combination of four pollutants—formaldehyde, carbon tetrachloride, acetaldehyde, and benzene (Table 2). Of the binary mixtures, carbon tetrachloride and formaldehyde; acetaldehyde and carbon tetrachloride; acetaldehyde and formaldehyde; and benzene and formaldehyde; benzene and carbon tetrachloride; acetaldehyde and benzene are found in a majority (>98%) of the census tracts (Table 2). Similarly, the ternary mixtures of acetaldehyde, carbon tetrachloride, and formaldehyde; benzene, carbon tetrachloride, and formaldehyde; acetaldehyde, benzene and carbon tetrachloride; and acetaldehyde, benzene and formaldehyde are found in a majority (> 98%) of the tracts.

Three basic types of approaches that vary in accuracy and uncertainties often are employed to make the most of available information and perform mixtures risk assessments: whole-mixtures approaches, similar-mixtures approaches, and component-based approaches [16]. There was no joint toxicology testing data found for the binary and ternary combinations of carcinogenic air toxics identified above. No toxicologically similar mixture can be justified as a surrogate. Thus, the only option left for this analysis is the component-based approach. Two general types of additivity are recognized in the component-based approach: dose additivity and response additivity [16,17]. Response additivity is often used for the carcinogenic risk assessment [18], which was used in NATA 2005 as well as in our health impact assessment. When more data on the joint toxicity of chemicals become available, the integration of potential interactions to estimate the increase or decrease of the joint toxicity could be considered in future risk assessments [19].

NATA 2005 shows that formaldehyde has a cancer risk of 22.5 in a million. The total national average cancer risk (for all 81 air toxics included in NATA 2005 assessment) is 50 in a million. Therefore, formaldehyde accounts for 45% of the national average risk in NATA 2005 assessment. In other words, among the 81 carcinogenic air toxics NATA assessed, formaldehyde alone accounts for nearly half of the total cancer risk. In addition, formaldehyde showed up consistently in our analysis regardless of urban vs. rural environment or the risk threshold we tested. Furthermore, formaldehyde alone accounts for 30 percent of the total cancer-related health impacts for the top 10 carcinogenic air toxics.

After formaldehyde, benzene accounts for the second most cancer related health impacts, together these two chemicals account for about 60% of the total cancer-related health impacts. Benzene also consistently shows up in our analysis in various binary and ternary combinations (Table 2). Apart from cancer, benzene is also known to cause several systemic health effects including hematological, immunological, and the central nervous system.

Given the key role of formaldehyde [20] and benzene [21] among the different carcinogenic air toxics mixtures, future research likely should investigate further their joint cancer potency between these two air toxics as well as with co-occurring chemicals such as carbon tetrachloride [22] and acetaldehyde [23].

We found some interesting comparisons between the urban and rural areas. The top four air toxics, i.e., carbon tetrachloride, formaldehyde, acetaldehyde, benzene, which had cancer risks greater than one in a million in more than 90% of the census tracts, are the same for urban and rural areas. Carbon tetrachloride is a long range transport pollutant. It is stable in the air with long atmospheric lifetime, estimated to range from 30 to 100 years [24]. In addition, there is little local emission. As a result, there is not much difference between urban and rural areas. For formaldehyde and acetaldehyde, their dominant sources are photochemical reactions, with 80 and 90 percent of their concentrations coming from secondary formations respectively, according to NATA 2005 data [25]. Their precursors come from both anthropogenic and natural sources such as trees, so that their concentrations can be high in both urban and rural locations. Outdoor benzene is from a variety of sources and its concentration can be high in both urban and rural areas due to emissions from different sources such as motor vehicle exhaust in urban areas, and fires and wood heaters in rural areas.

No other air toxics had cancer risk greater than one in a million in more than 25% of the census tracts in the rural areas. In comparison, there were several additional combinations found in more than 50% of the census tracts in the urban areas that included other air toxics e.g., 1,3- butadiene, naphthalene, arsenic and chromium compounds. This indicates that urban populations are exposed to a broader variety of air toxics compared to the rural populations.

The worldwide DALYs in 2010 attributable to outdoor PM_2.5_ and ozone were estimated to be 76 million and 2 million, respectively [26], which translate to an average of about 1,000 DALYs per 100,000 persons and 30 DALYs per 100,000 persons, respectively. In comparison, our results show that the cumulative annual carcinogenic health impact is less than 0.6 DALY per 100,000 persons from inhaling the top 10 carcinogenic air toxics in the outdoor air of the United States. This is more than three orders of magnitude smaller than the health impacts caused by PM_2.5_ and almost two orders of magnitude smaller than that caused by ozone on the global scale.

Several factors could have contributed to the difference in health impacts between the two criteria air pollutants and air toxics. First, we compared the worldwide health impacts of PM_2.5_ and ozone to the cancer-related health impacts of air toxics in the United States. Since some developing countries have significantly higher PM_2.5_ and ozone levels as well as much higher population density than the United States, the difference is likely an overestimate of the actual difference in the Unites States between criteria air pollutants and air toxics. Second, some of the carcinogenic air toxics under study are also known to cause non-carcinogenic health effects. Our current study focuses on carcinogenic health damage and therefore is an underestimate of the total health impacts. Thirdly, the methods for calculating the health impacts of PM_2.5_ and ozone are not directly comparable to that used for air toxics. For example, the annual health impacts of elevated PM_2.5_ and ozone levels are usually calculated by multiplying the annual background mortality rate, with population, with the increase in mortality rate per unit increase in pollutant concentration based on epidemiology study findings, and with the difference in actual pollutant concentration and the reference level. For air toxics, background mortality rate is not involved in the cancer-related health impacts estimates. The increase in lifetime cancer risk was first calculated by combining exposure concentration with inhalation unit risk estimate obtained from either epidemiology studies or extrapolation from animal studies. Then the annual increase in cancer risk was calculated by dividing the lifetime cancer risk with the expected lifetime, e.g., 70 years. The annual increase in cancer risk was then combined with other factors, e.g., population, cancer severity factor as discussed in more detail in Methods to calculate annual cancer-related health impacts. These difference in methods and data source could contribute to the difference in health impacts of these two types of air pollutants as well.

These differences in health impacts between criteria air pollutants (i.e., PM_2.5_ and ozone) and air toxics suggests that at the national level it might be more efficient to control air toxics with a co-benefit of reducing PM_2.5_ and/or ozone concentrations.

There are certain limitations to our study that are inherent to NATA analysis. First, our analysis is based on the results of the 2005 NATA data, which depended on emission inventory data and various models instead of monitoring data. For each combination of source type and air toxic, NATA 2005 assumes that all subjects in a census tract have the same exposure. EPA analyzed the model performance of the 2005 NATA through a model-to-monitor comparison. In this comparison, EPA calculated receptor-level concentrations from the NATA model and compared them to 2005 annual average concentrations of individual HAPs for several hundred air toxics monitoring sites across the country [27]. EPA found that ≈ 9% of all model-to-monitor ratios were within 10% (i.e., ratios between 0.9 and 1.1), 17% were within 20% (ratios between 0.8 and 1.2), and 25% were within 30% (ratios between 0.7 and 1.3). The top four pollutants, shown in our study, with cancer risk greater than one in a million (Table 2) are found to have good agreement with monitoring data. Comparisons have also been made between modeled concentrations from previous NATA assessments and monitoring data in different parts of the United States [28–30]. Overall, these studies found that NATA performance varied widely for different air toxics, though the predicted concentrations were generally within a factor of two of measured values for air toxics that were estimated to be the primary cancer-risk drivers.

Second, in terms of cancer-related health impacts, the DALY estimates for each cancer type was based on dated data [14]. Equal weightings for the importance of one year of life lost for all ages were assumed and no discounting for future damages were applied. Therefore, the cancer-related health impacts reported here would be higher than those reported by a similar study which applied discounting for future damages. The cancer-related health impact calculations were based on the selected cancer type as shown in Table 1, based on our review of toxicity databases such as IRIS, OEHHA Toxicity Criteria Database. However, an air toxic may cause more than one type of cancer. We currently can not quantitatively estimate the cancer-related health impact of the combined cancer types due to data constraints, as we do not have UREs for other cancer types. In addition, Polycyclic Aromatic Hydrocarbon and Polycyclic Organic Matter, or PAHPOM is made up of many air toxics. We calculated their cancer-related health impacts by using the cancer severity factor of lung cancer for this group of air toxics. Furthermore, some of the carcinogenic air toxics under study are also known to cause non-carcinogenic health effects through inhalation. The health impacts we reported in Table 2 are only the carcinogenic health damage and is therefore an underestimate of the total health impacts from the air toxics in the analysis.

Third, our study focused on the air toxics that affect public health most significantly at the national level, based on the frequency of air toxics mixtures and public health impacts. This analysis was not meant to provide a full picture of the locations of concern and a list of air toxics that contribute most substantially at each location. There could be air toxics which were not included in this study, since they caused significant cancer risks and negative health impacts only in a limited number of locations (e.g., less than 5% of census tracts).

## Conclusion

We have demonstrated that large databases such as NATA can be used to identify critical air toxics and their combinations. We identified several cancer causing air toxics combinations that show up consistently in a majority of census tracts regardless of urban vs. rural environment or the risk threshold, such as benzene, formaldehyde and acetaldehyde. These types of analyses and interpretations provide a realistic estimate of the levels of chemicals and their mixtures that occur in the environment. We believe this approach can be fine-tuned and used to identify and recommend specific mixtures for toxicity testing for various endpoints and health effects. Programs such as TOXCAST and TOX 21 [31,32] can be used to advance the understanding of priority chemical mixtures’ action mechanisms. A better understanding of the joint toxicity and cumulative health impacts of air toxics mixtures could facilitate identifying optimal environmental surveillance and control strategies to minimize the negative health impacts of these mixtures.

## Supporting Information

S1 File Air toxics with cancer risk reported in the National-Scale Air Toxics Assessment 2005 (Table A). Percent of census tracts with cancer risk greater than one in a million for single air toxic, binary pair, and ternary combination in urban census tracts (**Table B**). Percent of census tracts with cancer risk greater than one in a million for single air toxics, binary pairs, and ternary combinations in rural census tracts (**Table C**). Carcinogenic health impacts of formaldehyde in DALYs by census tract (**Figure A**). Carcinogenic health impacts of benzene in DALYs by census tract (**Figure B**).
(DOCX)
