# Supplementary material for: Carcinogenic Air Toxics Exposure and Their Cancer-Related Health Impacts in the United States
Source: PLoS One. 2015 Oct 7;10(10):e0140013. doi: 10.1371/journal.pone.0140013 (PMC4596837; doi:10.1371/journal.pone.0140013)

**Supporting Information**

Table A. Air toxics with cancer risk reported in the National-Scale Air Toxics Assessment 2005

| **Rank** | **air toxics** | **National average cancer risk** | **% tracts with cancer risk > one in a million** |
| --- | --- | --- | --- |
| 1 | formaldehyde | 2.2E-05 | 100.0% |
| 2 | benzene (including benzene from gasoline) | 7.6E-06 | 98.8% |
| 3 | acetaldehyde | 3.3E-06 | 99.8% |
| 4 | carbon tetrachloride | 2.9E-06 | 100.0% |
| 5 | naphthalene | 2.3E-06 | 62.4% |
| 6 | 1,3-butadiene | 2.0E-06 | 70.7% |
| 7 | PAHPOM | 1.5E-06 | 40.4% |
| 8 | chromium compounds | 1.4E-06 | 51.0% |
| 9 | arsenic compounds (inorganic including arsine) | 1.4E-06 | 53.4% |
| 10 | tetrachloroethylene (perchloroethylene) | 1.1E-06 | 33.4% |
| 11 | 1,4-dichlorobenzene | 8.4E-07 | 25.4% |
| 12 | Ethylbenzene | 6.5E-07 | 20.6% |
| 13 | ethylene oxide | 5.6E-07 | 13.6% |
| 14 | Acrylonitrile | 3.9E-07 | 7.2% |
| 15 | nickel compounds | 2.4E-07 | 3.8% |
| 16 | coke oven emissions | 2.2E-07 | 4.0% |
| 17 | ethylene dibromide (dibromoethane) | 2.2E-07 | 0.1% |
| 18 | 1,1,2,2-tetrachloroethane | 1.8E-07 | 0.3% |
| 19 | methylene chloride (dichloromethane) | 1.5E-07 | 0.6% |
| 20 | 1,3-dichloropropene | 1.5E-07 | 3.1% |
| 21 | Trichloroethylene | 1.3E-07 | 0.2% |
| 22 | cadmium compounds | 1.2E-07 | 0.4% |
| 23 | ethylene dichloride (1,2-dichloroethane) | 8.3E-08 | 0.2% |
| 24 | methyl tert-butyl ether | 7.9E-08 | 0.2% |
| 25 | beryllium compounds | 7.8E-08 | 0.2% |
| 26 | Hydrazine | 3.2E-08 | 0.1% |
| 27 | vinyl chloride | 3.0E-08 | 0.1% |
| 28 | propylene dichloride (1,2-dichloropropane) | 2.5E-08 | 0.0% |
| 29 | Benzidine | 1.4E-08 | 0.1% |
| 30 | benzotrichloride | 1.2E-08 | 0.2% |
| 31 | n-nitrosomorpholine | 1.2E-08 | 0.2% |
| 32 | bis(2-ethylhexyl)phthalate (DEHP) | 9.4E-09 | 0.0% |
| 33 | 1,1,2-trichloroethane | 9.1E-09 | 0.2% |
| 34 | 1,2-dibromo-3-chloropropane | 5.9E-09 | 0.0% |
| 35 | 4,4'-methylene bis(2-chloroaniline) | 2.6E-09 | 0.0% |
| 36 | 4,4'-methylenedianiline | 2.4E-09 | 0.0% |
| 37 | propylene oxide | 2.1E-09 | 0.0% |
| 38 | 2,4-toluene diisocyanate | 2.0E-09 | 0.0% |
| 39 | polychlorinated biphenyls (aroclors) | 1.5E-09 | 0.0% |
| 40 | 2,4-dinitrotoluene | 1.4E-09 | 0.0% |
| 41 | benzyl chloride | 1.2E-09 | 0.0% |
| 42 | ethylidene dichloride (1,1-dichloroethane) | 1.2E-09 | 0.0% |
| 43 | ethyl carbamate (urethane) chloride (chloroethane) | 1.1E-09 | 0.0% |
| 44 | acrylamide | 1.0E-09 | 0.0% |
| 45 | 1,4-dioxane | 9.1E-10 | 0.0% |
| 46 | 2,4-toluene diamine | 8.5E-10 | 0.0% |
| 47 | hexachlorobenzene | 6.8E-10 | 0.0% |
| 48 | nitrobenzene | 3.5E-10 | 0.0% |
| 49 | 1,3-propane sultone | 3.5E-10 | 0.0% |
| 50 | 1,2,3,4,5,6-hexachlorocyclyhexane (all stereo isomers) | 2.8E-10 | 0.0% |
| 51 | 4-dimethylaminoazobenzene | 2.8E-10 | 0.0% |
| 52 | pentachloronitrobenzene (quintobenzene) | 2.5E-10 | 0.0% |
| 53 | dichloroethyl ether (bis[2-chloroethyl]ether) | 1.9E-10 | 0.0% |
| 54 | epichlorohydrin | 1.8E-10 | 0.0% |
| 55 | o-toluidine | 1.8E-10 | 0.0% |
| 56 | 2-nitropropane | 1.6E-10 | 0.0% |
| 57 | Aniline | 1.5E-10 | 0.0% |
| 58 | trifluralin | 1.3E-10 | 0.0% |
| 59 | allyl chloride | 1.2E-10 | 0.0% |
| 60 | bis(chloromethyl) ether | 7.4E-11 | 0.0% |
| 61 | isophorone | 7.2E-11 | 0.0% |
| 62 | dichlorvos | 6.9E-11 | 0.0% |
| 63 | hexachlorobutadiene | 6.4E-11 | 0.0% |
| 64 | acetamide | 5.1E-11 | 0.0% |
| 65 | n-nitrosodimethylamine | 3.9E-11 | 0.0% |
| 66 | hexachloroethane | 3.4E-11 | 0.0% |
| 67 | pentachlorophenol | 1.4E-11 | 0.0% |
| 68 | ethylene thiourea | 6.4E-12 | 0.0% |
| 69 | bromoform | 2.7E-12 | 0.0% |
| 70 | heptachlor | 2.0E-12 | 0.0% |
| 71 | vinyl bromide | 1.5E-12 | 0.0% |
| 72 | captan | 1.3E-12 | 0.0% |
| 73 | chlorobenzilate | 1.3E-12 | 0.0% |
| 74 | 3,3'-dimethylbenzidine | 6.9E-13 | 0.0% |
| 75 | 2,4,6-trichlorophenol | 6.6E-13 | 0.0% |
| 76 | toxaphene (chlorinated camphene) | 5.9E-13 | 0.0% |
| 77 | Chlordane | 5.3E-13 | 0.0% |
| 78 | 3,3'-dichlorobenzidine | 4.0E-13 | 0.0% |
| 79 | DDE (1,1-dichloro-2,2-bis(p- chlorophenyl) ethylene) | 1.8E-13 | 0.0% |
| 80 | 3,3'-dimethoxybenzidine | 1.3E-15 | 0.0% |
| 81 | cyanide compounds | 0.0E+00 | 0.0% |

Note: Rank is based on the average cancer risk at the national level

Table B. Percent of census tracts with cancer risk greater than one in a million for single air toxic, binary pair, and ternary combination in urban census tracts

| **Single air toxic** | | | | **Binary Pair** | | | **Ternary Combination** | | | |  |
| --- | --- | --- | --- | --- | --- | --- | --- | --- | --- | --- | --- |
| **Rank** | **Air Toxic** | | **Percent of Census tracts^*^** | **Rank** | **Air Toxic** | **Percent of Census tracts^**^** | **Rank** | **Air Toxic** | | **Percent of Census tracts^***^** |  |
| 1 | formaldehyde | | 100.0% | 1 | carbon tetrachloride, formaldehyde | 100.0% | 1 | benzene, carbon tetrachloride, formaldehyde | | 100.0% |  |
| 2 | carbon tetrachloride | | 100.0% | 2 | benzene, formaldehyde | 100.0% | 2 | acetaldehyde, carbon tetrachloride, formaldehyde | | 99.9% |  |
| 3 | benzene | | 100.0% | 3 | benzene, carbon tetrachloride | 100.0% | 3 | acetaldehyde, benzene, formaldehyde | | 99.9% |  |
| 4 | acetaldehyde | | 99.9% | 4 | acetaldehyde, formaldehyde | 99.9% | 4 | acetaldehyde, benzene, carbon tetrachloride | | 99.9% |  |
| 5 | 1,3-butadiene | | 85.1% | 5 | acetaldehyde, carbon tetrachloride | 99.9% | 5 | 1,3-butadiene, benzene, formaldehyde | | 85.1% |  |
| 6 | naphthalene | | 74.0% | 6 | acetaldehyde, benzene, | 99.9% | 6 | 1,3-butadiene, carbon tetrachloride, formaldehyde | | 85.1% |  |
| 7 | arsenic compounds | | 63.1% | 7 | 1,3-butadiene, formaldehyde | 85.1% | 7 | 1,3-butadiene, benzene, carbon tetrachloride | | 85.1% |  |
| 8 | chromium compounds | | 60.8% | 8 | 1,3-butadiene, benzene | 85.1% | 8 | 1,3-butadiene, acetaldehyde, formaldehyde | | 85.1% |  |
| 9 | PAHPOM | | 44.6% | 9 | 1,3-butadiene, carbon tetrachloride | 85.1% | 9 | 1,3-butadiene, acetaldehyde, carbon tetrachloride | | 85.1% |  |
| 10 | tetrachloroethylene | | 40.2% | 10 | 1,3-butadiene, acetaldehyde | 85.1% | 10 | 1,3-butadiene, acetaldehyde, benzene | | 85.1% |  |
| Note: | | | | | | | | |  | | |
| * Percent of census tracts with cancer risk above one in a million for the corresponding air toxic | | | | | | | | |  | | |
| ** Percent of census tracts with cancer risk above one in a million for both air toxics in the pair | | | | | | | | | | | |
| *** Percent of census tracts with cancer risk above one in a million for all three air toxics in the combination | | | | | | | | | | | |
|  | |  |  |  |  |  |  |  |  |  |  |

Table C. Percent of census tracts with cancer risk greater than one in a million for single air toxics, binary pairs, and ternary combinations in rural census tracts

| **Single air toxic** | | | **Binary Pair** | | | **Ternary Combination** | | | |  |
| --- | --- | --- | --- | --- | --- | --- | --- | --- | --- | --- |
| **Rank** | **Air Toxic** | **Percent of Census tracts^*^** | **Rank** | **Air Toxic** | **Percent of Census tracts^**^** | **Rank** | **Air Toxic** | | **Percent of Census tracts^***^** |  |
| 1 | carbon tetrachloride | 100.0% | 1 | carbon tetrachloride, formaldehyde | 100.0% | 1 | acetaldehyde, carbon tetrachloride, formaldehyde | | 99.4% |  |
| 2 | formaldehyde | 100.0% | 2 | acetaldehyde, carbon tetrachloride | 99.4% | 2 | benzene, carbon tetrachloride, formaldehyde | | 94.0% |  |
| 3 | acetaldehyde | 99.4% | 3 | acetaldehyde, formaldehyde | 99.4% | 3 | acetaldehyde, benzene, carbon tetrachloride | | 93.5% |  |
| 4 | benzene | 94.0% | 4 | benzene, carbon tetrachloride | 94.0% | 4 | acetaldehyde, benzene, formaldehyde | | 93.5% |  |
| 5 | PAHPOM | 22.6% | 5 | benzene, formaldehyde | 94.0% | 5 | carbon tetrachloride, formaldehyde, PAHPOM | | 22.6% |  |
| 6 | naphthalene | 12.9% | 6 | acetaldehyde, benzene | 93.5% | 6 | benzene, carbon tetrachloride, PAHPOM | | 22.6% |  |
| 7 | arsenic compounds | 12.3% | 7 | carbon tetrachloride, PAHPOM | 22.6% | 7 | benzene, formaldehyde, PAHPOM | | 22.6% |  |
| 8 | 1,3-butadiene | 10.0% | 8 | formaldehyde, PAHPOM | 22.6% | 8 | acetaldehyde, carbon tetrachloride, PAHPOM | | 22.6% |  |
| 9 | chromium compounds | 9.0% | 9 | benzene, PAHPOM | 22.6% | 9 | acetaldehyde, formaldehyde, PAHPOM | | 22.6% |  |
| 10 | tetrachloroethylene | 4.5% | 10 | acetaldehyde, PAHPOM | 22.6% | 10 | acetaldehyde, benzene, PAHPOM | | 22.6% |  |
| Note: | | | | | | | |  | | |
| * Percent of census tracts with cancer risk above one in a million for the corresponding air toxic | | | | | | | |  | | |
| ** Percent of census tracts with cancer risk above one in a million for both air toxics in the pair | | | | | | | | | | |
| *** Percent of census tracts with cancer risk above one in a million for all three air toxics in the combination | | | | | | | | | | |

Figure A. Carcinogenic health impacts of formaldehyde in DALYs by census tract


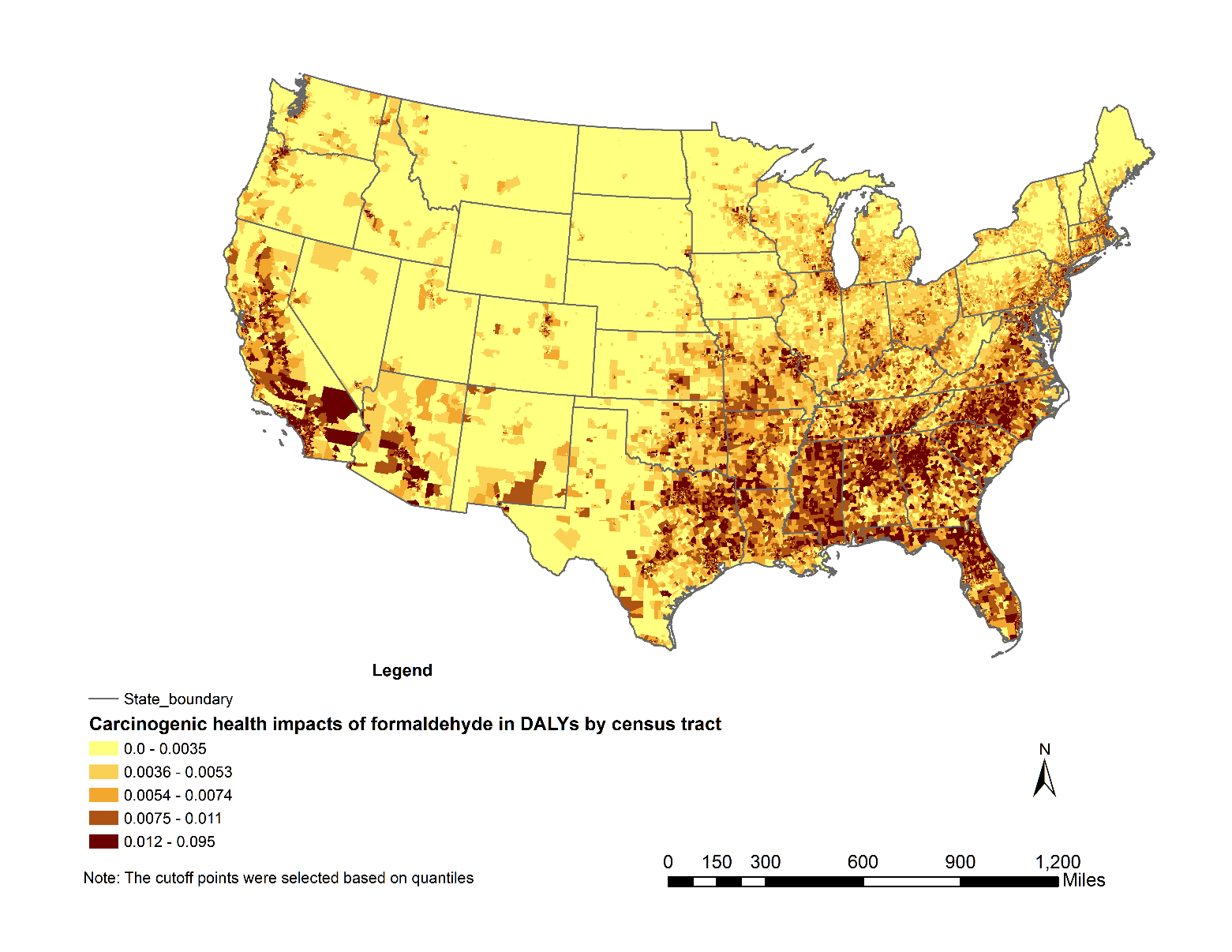


Figure B. Carcinogenic health impacts of benzene in DALYs by census tract


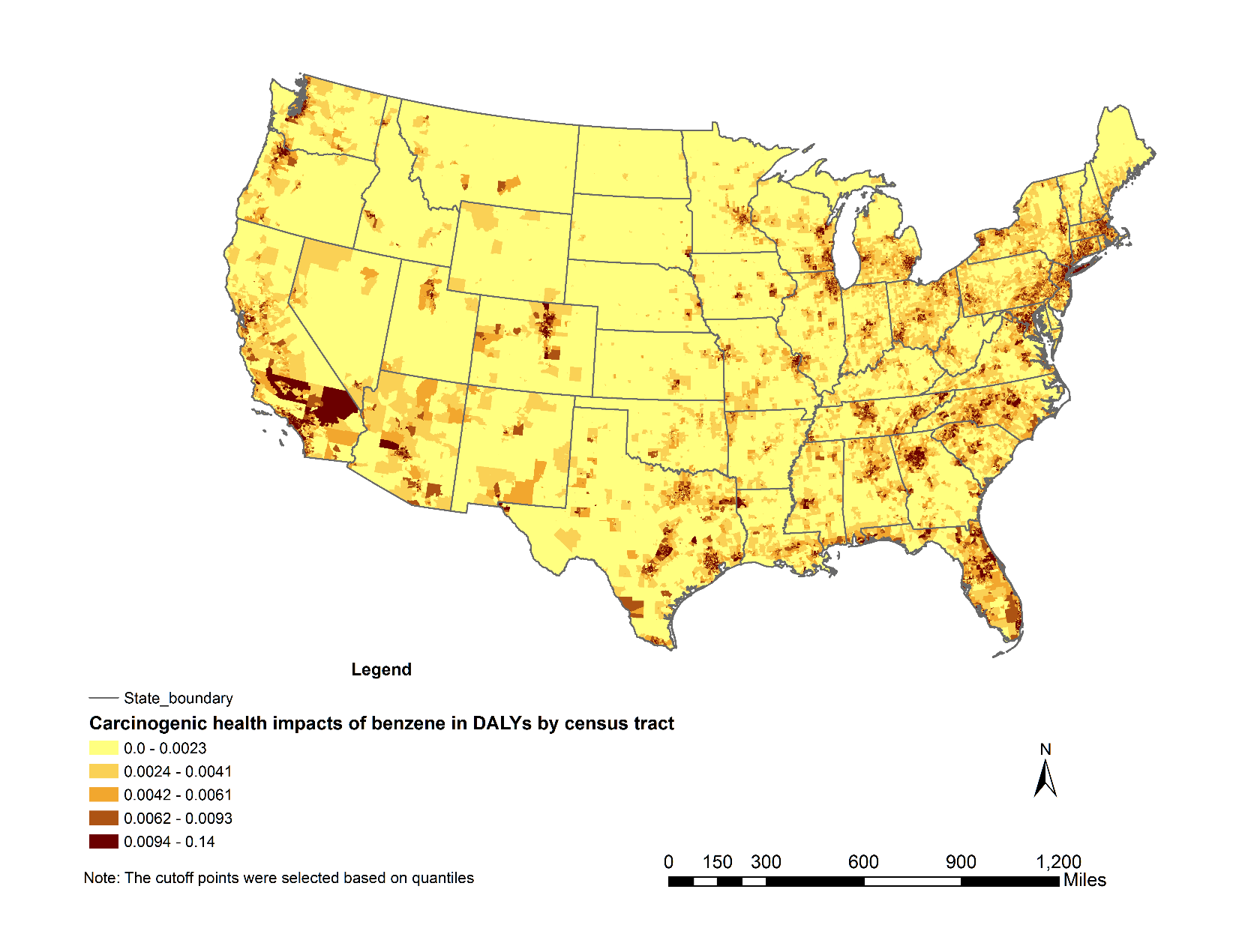

Supplement: S1 File — Percent of census tracts with cancer risk greater than one in a million for single air toxic, binary pair, and ternary combination in urban census tracts (Table B). Percent of census tracts with cancer risk greater than one in a million for single air toxics, binary pairs, and ternary combinations in rural census tracts (Table C). Carcinogenic health impacts of formaldehyde in DALYs by census tract (Figure A). Carcinogenic health impacts of benzene in DALYs by census tract (Figure B). (DOCX) [file pone.0140013.s001.docx]
